# Hepatic larva migrans presenting with upper gastrointestinal haemorrhage: A case report

**DOI:** 10.4102/sajr.v25i1.2200

**Published:** 2021-11-26

**Authors:** Ranjan K. Patel, Shruti Mittal

**Affiliations:** 1 Department of Interventional Radiology, Institute of Liver and Biliary Sciences, New Delhi, India; 2 Department of Radiodiagnosis, Maulana Azad Medical College, New Delhi, India

**Keywords:** visceral larva migrans (VLM), hepatic larva migrans, pseudoaneurysm, hepatic artery, portal vein thrombosis, coil embolisation

## Abstract

Visceral larva migrans (VLM) occurs because of a host inflammatory response to the migrating larvae of a nematode. Patients usually present with fever, hepatomegaly and abdominal pain; vascular arterial complications are uncommon. A 19-year female presented with fever, jaundice, abdominal discomfort and melena. Computed tomography (CT) revealed multiple discrete, clustered, complex hepatic cystic lesions consistent with VLM, along with an arterial pseudoaneurysm from the right hepatic artery which was managed with endovascular coil embolisation.

## Introduction

Visceral larva migrans (VLM) results from the migratory larva of nematodes, with the lungs and liver being the most common organs involved. Presentation varies depending on the site of involvement.^1,2^ Pulmonary involvement manifests as fever, cough, wheezing and chest pain, whereas hepatic involvement presents with fever, hepatomegaly and abdominal pain. Hepatic larva migrans presenting with gastrointestinal bleeding is very uncommon.^2^ This report describes a case of hepatic larva migrans in a 19-year female, manifesting as upper gastrointestinal (GI) bleeding secondary to a hepatic artery pseudoaneurysm, which was successfully managed with endovascular coil embolisation.

## Case history

**FIGURE 1:**
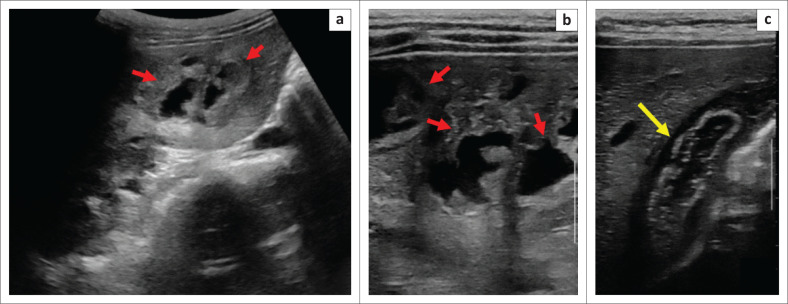
Ultrasonography of the liver. Image (a and b) showing multiple clustered hypo and anechoic complex cystic lesions (red arrows) with a mildly hyperechoic rim. Image (c) showing an oedematous gall bladder wall (yellow arrow).

A 19-year-old female presented with complaints of intermittent low-grade fever for two months, associated with right-sided mild upper abdominal discomfort, yellowish discolouration of the sclera and a history of black-coloured stool for 7–10 days. The patient belonged to a lower socio-economic status and was living in an overcrowded area. There was no obvious history of exposure to any pet animals. On examination, she was afebrile and hemodynamically stable with a pulse rate of 80 beats/min and a blood pressure of 112/64 millimeter of mercury (mmHg). She also had pallor and icterus. Abdominal examination revealed mildly tender hepatomegaly. The rest of the systemic examination findings were unremarkable. The relevant laboratory parameters were: low haemoglobin (6.1 gm/dL), leucocytosis (14 200/mm^3^), eosinophilia (1.26 × 10^9^/L), mildly elevated bilirubin (total −1.7 mg/dL, direct −1.3 mg/dL), elevated alkaline phosphatase (620 IU/L) and gamma-glutamyl transferase (364 IU/L); the remaining results were within normal limits. There was no previous history of any hepatic intervention. Emergency ultrasonography of the abdomen revealed multiple discrete and clustered hypo to anechoic lesions with a mildly hyperechoic irregular rim in the right lobe of the liver and mildly prominent central biliary radicles (Figure 1a, b). The gall bladder was mildly distended with an oedematous wall (Figure 1c). No obvious hematoma or pseudoaneurysm was found at ultrasonography (USG). The patient was started on empiric broad-spectrum intravenous antibiotics and transfused two units of blood.

**FIGURE 2:**
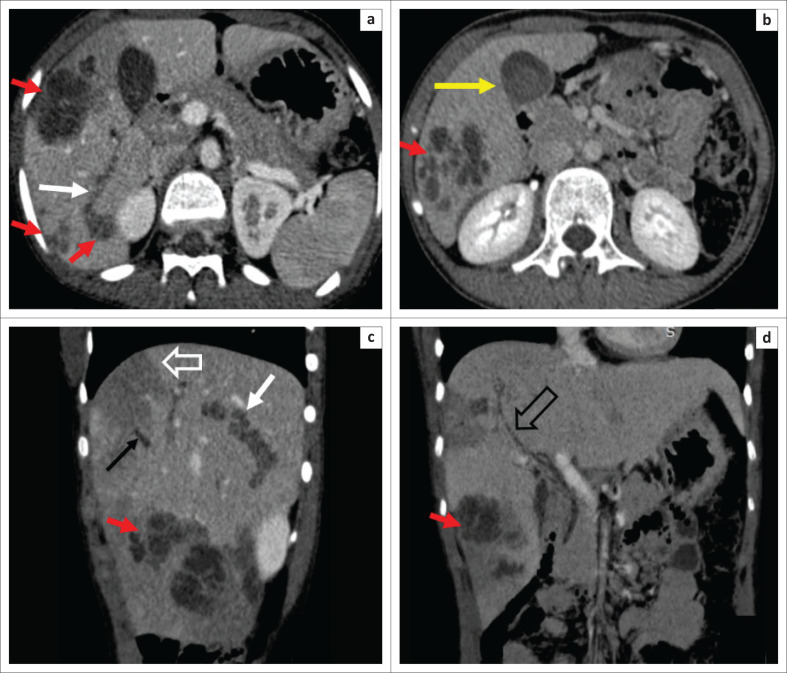
Axial (a, b) and sagittal (c) portal venous phase CT images showing multiple clustered hypodense lesions (red arrows) in the right lobe of the liver with indistinct margins. Few linear hypodense tracks (white arrows a, c) are also seen, consistent with the migration of larvae. There is hyperdense content within the gall bladder (GB) lumen (yellow arrow b) sludge or haemorrhage. A small non-enhancing portal vein (PV) segment is noted on sagittal image c in segment VIII, suggestive of segmental PV thrombosis (thin black arrow c). Associated transient hepatic arterial difference (THAD) is also found (open white arrow c). Coronal minimum intensity projection (MinIP) image (d) showing mildly dilated central biliary radicles (open black arrow).

**FIGURE 3:**
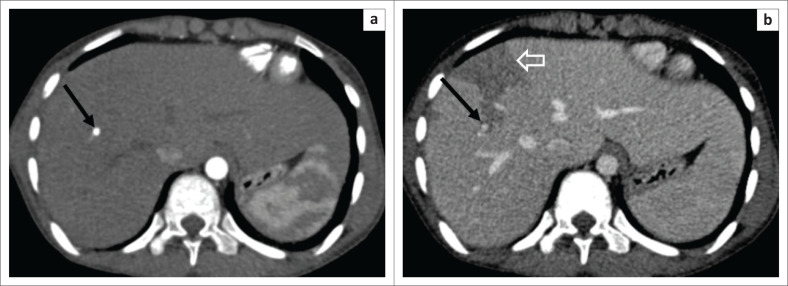
Axial arterial phase (a) image showing a well-defined contrast pooling (black arrow) in segment VIII with its attenuation parallel to that of the abdominal aorta without any change in morphology as seen in the axial portal venous phase image (black arrow b). It remains isodense to the aorta in the portal venous phase as well, suggestive of hepatic arterial pseudoaneurysm without any apparent active contrast extravasation. A geographical peripheral hypodense area is noted in segment VIII (open white arrow b), representing a transient hepatic arterial difference (THAD).

Dual-phase computed tomography (CT) of the abdomen revealed multiple discrete and clustered variable-sized hypoechoic lesions with an enhancing rim in the right lobe of the liver, predominantly along the peripheral hepatic parenchyma (Figure 2). A few linear hypodense tracks were also depicted, consistent with the migration of larvae (Figure 2a and 2c). Small segmental portal thrombosis was found in segment VIII along with a few geographical areas of hypoperfusion noted as hypodensity on the portal-venous phase (Figure 2c). Mildly dilated biliary radicles were demonstrated on the minimum intensity projection (MinIP) images (Figure 2d). Additionally, there was a well-defined pseudoaneurysm of 1.1 cm size along the anterior branch of the right hepatic artery; however, no apparent active contrast extravasation was seen (Figure 3a, b).

**FIGURE 4:**
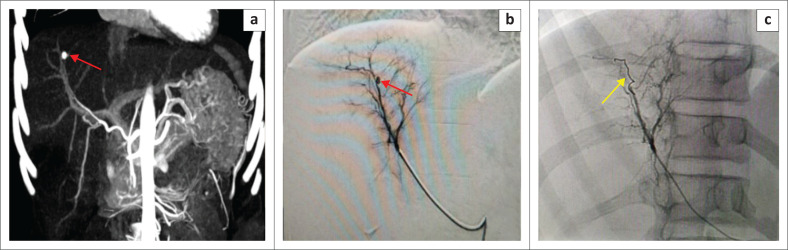
Coronal CT maximum intensity projection image (a) shows a well-defined pseudoaneurysm in close proximity to the segment VIII artery (red arrow a). Angiography via a microcatheter (b) shows a small pseudoaneurysm arising from the segment VIII artery (red arrow b) which was embolised (c) by placing a microcoil across the neck of pseudoaneurysm using the sandwich technique (yellow arrow c).

Subsequent upper GI endoscopy showed haemobilia without any varices. Thus, pseudoaneurysm was suspected to be the cause for haemobilia. The branch of the right hepatic artery from which pseudoaneurysm originated was selectively catheterised with a microcatheter through a right femoral artery approach and the pseudoaneurysm was embolised with microcoils using a sandwich technique (Figure 4).

The following differential diagnoses were considered in view of the clinical and imaging findings: pyogenic liver abscesses, VLM, fascioliasis, cat-scratch disease, disseminated tuberculosis and atypical hydatid disease. Further investigations revealed positive toxocara immunoglobulin G (IgG) serology. Stool examination, hydatid serology Mantoux test were negative. Fluid aspirated from the lesions was negative for bacterial and fungal pathogens. Histopathological examination of fine needle aspiration showed eosinophilic abscesses with poorly defined eosinophilic granulomas and Charcot-Leyden crystals, suggestive of a parasitic infection.

Based on clinical, imaging and pathological findings with a positive toxocara IgG serology, a diagnosis of hepatic larva migrans was made and the patient was treated with albendazole (400 mg twice a day for two weeks). Oral prednisone (20 mg/day) was also added for one week and tapered gradually. The patient was discharged in a stable condition after the resolution of fever and abdominal pain. There was no sign of any further GI bleeding. Follow-up imaging was not available; however, she was clinically fine at six months follow-up on teleconsultation.

## Discussion

Visceral larva migrans, also known as toxocariasis, represents the migratory phase of the 2nd stage larvae of nematodes through the different visceral organs. *Toxocara canis* is the most common causative organism. The other less common causative organisms include *Toxocara catis, Baylisascaris procyonis, Capillaria hepatica, Ascaris sum* and some *Ancylostoma* species.^1^ As humans are not the definitive host, larvae cannot mature and continue migrating through the different visceral organs for months to years. These migratory larvae initiate a host inflammatory response along with eosinophilic infiltration, resulting in tissue destruction and variable clinical manifestations.^3^ Visceral larva migrans is more common in children.^4^ The liver and lungs are the most common sites of involvement.^1,4^ As seen in this case, hepatic involvement manifests as fever, hepatomegaly, decrease in appetite and abdominal pain.

There is no characteristic imaging feature of VLM; however, some features on imaging are suggestive, and it often needs correlation with laboratory and pathological findings.^5,6,7^ Imaging findings reflect the pathological process. Periportal eosinophilic infiltration, granulomas and abscesses with central necrosis caused by migration of larvae appear as multiple variable-sized, ill-defined discrete and conglomerating, oval or elongated focal lesions on imaging.^3,5,7,8^ Lesions are hypoechoic on ultrasonography. They are best depicted on portal venous phase CT images, appearing as hypodense discrete and clustered complex cystic lesions, likely along the portal vein (PV) branches or liver periphery.^5,6,9^ In larger lesions, the PV seems to traverse through the lesions.^9^ At MRI, lesions show variable signal intensities on T1 and T2 weighted images. A T1 hyperintense rim around the abscess cavity may be an important imaging feature, indicating a layer of granulation tissue and thus the inflammatory nature of the lesion. The abscess cavity shows variable diffusion restriction.^5,6,7^ Lesions may show a change in morphology and position on follow-up imaging, a finding consistent with the migration of larvae. Sometimes, linear tracts are also seen, as noted in this case, which could be a very useful imaging feature that raises a suspicion of VLM.^2^

Cytotoxic eosinophil-derived proteins may damage nearby vessels that lead to different vascular complications.^10^ Desai et al. reported two cases of VLM, complicated with PV thrombosis.^11^ Laroia et al. and Kaur et al. described hepatic vein thrombosis in patients with VLM.^5,12^ Segmental PV thrombosis was also seen in this case that resulted in sub-segmental geographical hypodensity in the portal-venous phase. Arterial complications are even rarer. Only one case has been reported to date by Ritu et al.^13^ They illustrated a case of VLM presenting with haemobilia because of a pseudoaneurysm of the right hepatic artery, which was embolised using N-butyl cyanoacrylate (NBCA) glue.^13^ Similarly, this case demonstrated a pseudoaneurysm arising from the segment VIII artery which was managed with coil embolisation.

New generation ELISA-based serological tests form the mainstay of diagnosis. Additional useful findings are the presence of eosinophilia and elevated immunoglobulin E (IgE) levels. Cytology or histology helps in excluding other differential diagnoses.^9^

Oral albendazole is the treatment of choice with concomitant steroid and antihistaminic therapy to reduce inflammation. However, long-term therapy is often needed, and in some cases, lobar or segmental hepatic resection is required depending on the extent of hepatic involvement.^5,7,13,14^

## Conclusion

Multiple ill-defined clustered lesions along the PV branches in the proper clinical background should raise the suspicion of VLM. Arterial pseudoaneurysm is an infrequent complication of VLM. Dual-phase CT is essential in the characterisation of lesions and detection of vascular complications. Emergency endovascular embolisation is an effective treatment in cases with a bleeding pseudoaneurysm.
